# Intraoperative parathyroid hormone monitoring in parathyroidectomy for hyperparathyroidism: a protocol for a network meta-analysis of diagnostic test accuracy

**DOI:** 10.3389/fsurg.2023.1298611

**Published:** 2024-01-04

**Authors:** Phillip Staibano, Kevin Um, Sheila Yu, Mohit Bhandari, Michael K. Gupta, Michael Au, JEM (Ted) Young, Han Zhang

**Affiliations:** ^1^ Division of Otolaryngology–Head and Neck Surgery, Department of Surgery, McMaster University, Hamilton, ON, Canada; ^2^ Department of Medicine, McMaster University, Hamilton, ON, Canada; ^3^ Department of Surgery, McMaster University, Hamilton, ON, Canada

**Keywords:** parathyroid, hyperparathyroidism, network meta-analysis, surgery, intraoperative parathyroid hormone (PTH) assay

## Abstract

Intraoperative parathyroid hormone (iPTH) monitoring is standard-of-care in the surgical management of hyperparathyroidism. It involves real-time determination of circulating PTH levels to guide parathyroid gland excision. There exists several iPTH monitoring criteria, such as the Miami criteria, and a lack of standardization in the timing of post-parathyroid gland excision samples. We present a protocol of a systematic review and network meta-analysis of diagnostic test accuracy to identify the iPTH criteria and post-gland excision timepoint that best predicts surgical cure in hyperparathyroidism. The database search strategy will be developed in conjunction with a librarian specialist. We will perform a search of Medline (Ovid), EMBASE (Ovid), CINAHL, Cochrane Collaboration, and Web of Science from 1990–present. Studies will be eligible if they include adult patients diagnosed with hyperparathyroidism who undergo parathyroidectomy with iPTH monitoring. We will only include studies that report diagnostic test properties for iPTH criteria and/or post-excision sampling timepoints. All screening, full-text review, data extraction, and critical appraisal will be performed in duplicate. Critical appraisal will be performed using QUADAS-2 instrument. A descriptive analysis will present study and critical appraisal characteristics. We will perform evaluation of between-study heterogeneity using *I*^2^ and Cochrane Q and where applicable, we will perform sensitivity analysis. Our network meta-analysis will include Bayesian hierarchical framework with random effects using multiple models. Ethics approval is not required. This proposed systematic review will utilize a novel Bayesian network meta-analysis model to help standardize iPTH monitoring in hyperparathyroidism, thereby optimizing patient outcomes and healthcare expenditures.

## Introduction

Hyperparathyroidism is characterized by the abnormal circulating levels of parathyroid hormone (PTH) and calcium that can lead to osteoporosis, renal calculi, and cognitive deficits (1). Primary hyperparathyroidism is often caused by a single hyperfunctioning parathyroid adenoma, but in 10%–15% of cases can be caused by double adenoma and/or parathyroid hyperplasia. Secondary and tertiary hyperparathyroidism, which are associated with chronic kidney disease are often secondary to parathyroid hyperplasia (2). In all cases, however, definitive cure requires surgical extirpation of the affects parathyroid gland(s). Advances in imaging continue to improve our ability to detect parathyroid lesions and therefore guide minimally invasive surgical approaches, but current imaging modalities remain inaccurate in detecting parathyroid hyperplasia (3, 4). Patients with discordant or negative imaging, in addition to those with suspected parathyroid hyperplasia, still often require bilateral neck exploration (5).

Intraoperative PTH (iPTH), which involves the real-time sampling of circulating PTH levels to guide parathyroidectomy, improves surgical outcomes and is standard-of-care for managing primary hyperparathyroidism (6, 7). Moreover, iPTH monitoring has high sensitivity in predicting surgical cure within renal hyperparathyroidism and may assist in identifying parathyroid carcinoma (8, 9). Several iPTH criteria exist to assist surgeons in standardizing changes in circulating parathyroid level during surgery, including the Miami criteria, which defines operative success as >50% decrease in iPTH from baseline value for all hyperfunctioning glands (10).

The primary goal of this systematic review and diagnostic test accuracy network meta-analysis will be to determine the optimal iPTH criteria and post-parathyroid gland excision sampling timepoint that best predict surgical cure in primary and renal hyperparathyroidism.

## Methods

### Protocol and registration

We will conduct a diagnostic test network meta-analysis to determine the iPTH criteria and post-parathyroid gland excision sampling timepoints most predictive of surgical cure. We registered the protocol with the Open Science Framework register of systematic reviews (OSF Registration; DOI: 10.17605/OSF.IO/4WEMJ). This protocol will be published under the guidelines of the Preferred Reporting Items for Systematic Reviews and Meta-Analysis for Protocols (PRISMA-P) (11). Any notable protocol amendments will be reported and published alongside the final review, which will be reported in accordance with the PRISMA statement (12).

### Study eligibility criteria

#### Population

We will include studies that investigate adult patients (≥18 years old) diagnosed with primary, secondary, and/or tertiary hyperparathyroidism who have undergone primary or revision parathyroidectomy via traditional midline neck incision or minimally invasive approach. There will be no further restrictions on the patient population.

#### Comparator (index test)

Our index test is the iPTH assay using any validated laboratory instrument. We will be evaluating any of the published iPTH criteria (e.g., Miami, Vienna, Rome, or Halle criteria) and/or the post-parathyroid gland excision sampling time points (e.g., 5-, 10-, 15-, 20-, and 25-min post-parathyroid gland ligation).

#### Reference standard

Our reference standard will be surgical cure following parathyroidectomy. We will define surgical cure as the resolution of hyperparathyroidism (i.e., resolution of hypercalcemia and/or hyperparathyroidism) within 3–6 months of parathyroidectomy.

#### Outcome

Our primary outcome will be centred around diagnostic test properties for all index tests as compared to the reference standard (e.g., pooled sensitivity, specificity, positive predictive value, negative predictive value, and likelihood ratios). We will also determine diagnostic odds ratio, post-test probabilities, and superiority index.

#### Study designs

We will consider studies eligible if they are randomized or non-randomized trials, cross-sectional, case-control, cohort studies, or case series with more than five patients. All studies must report study outcomes in a way that permits analysis of diagnostic test properties. There will be no restrictions on length of follow-up for longitudinal studies.

#### Language

Google Translate may be utilized for eligible articles published in non-English languages. This software, however, may not be appropriate for all languages, and so translators may be considered depending on the number of articles retrieved, in addition to the time and costs associated with each translation.

#### Study exclusions

We will exclude articles that study pediatric populations and those that do not report data in manner that permits extraction of diagnostic test properties.

### Information sources

We will perform a database search of PubMed (Ovid), EMBASE (Ovid), Cochrane Central Register of Controlled Trials, and Web of Science from January 1, 1990, to July 20, 2023. We will include studies published after 1990 since operative use of PTH was first described in 1991 (13). We will include human studies and will not place any language restrictions. We will also perform a search of reference lists of relevant systematic reviews, narrative reviews, and meta-analyses. We will not perform a search of the grey literature.

### Search strategy

We will include relevant search terms and MeSH headlines. All terms will be exploded when appropriate. We did evaluate the quality of the search by determining its ability to capture three pre-selected candidate articles. We will include the following example terms: primary, secondary, renal, and tertiary hyperparathyroidism, intraoperative parathyroid hormone monitoring, and iPTH criteria (e.g., Miami, Rome, Halle, etc.). The Medline search algorithm is presented in Appendix A.

### Data management

A single reviewer will implement the search strategy with the help of librarian specialist and manage all citations. These combined citations will be exported into an article management software, Covidence (Melbourne, Australia), which removes duplicate articles. All extracted data will be managed in a piloted, customized Excel (Redmond, Washington, USA) spreadsheet.

#### Selection process

We will begin screening with a pilot screening aid to assess agreement between two reviewers using 10% of the articles. We will define good agreement as a two-reviewer consensus rate of 75% based on the inclusion and exclusion decisions of reviewers. If this pilot identifies discrepancies of >75%, then we will consider modifying the inclusion criteria and report these protocol deviations in the final review.

#### Data extraction

We will pilot a tailored extraction form by evaluating five articles to assess agreement between the reviewers. This extraction pilot will be evaluated by third reviewer and if deemed congruent between reviewers, then extraction for all full-text articles will proceed. We will evaluate study characteristics (e.g., name of first author, year of publication, country, gold standard, index tests), patient characteristics (e.g., gender, mean age, sample, method, cut-off level, type of disease, type of surgery, details of preoperative workup), and outcomes (i.e., true positive, false positive, true negative, false negative). All final extraction information will be performed by two reviewers and evaluated by a third reviewer. We have included the extraction form in Appendix B.

#### Critical appraisal of individual articles (risk of bias)

Two reviewers will critically appraise the selected studies using the Quality Assessment of Diagnostic Accuracy Studies (QUADAS-2) instrument (14). All disagreements will be solved by consensus review between the two reviewers and/or by a third reviewer. The methodological quality of individual studies will be reported in the final text and tables.

### Data synthesis and analysis

#### Descriptive synthesis

A descriptive synthesis will present the characteristics of included studies, critical appraisal results, and descriptions of the main findings. Where applicable, the descriptive synthesis will be summarized according to each iPTH criteria and/or post-gland excision sampling timepoint.

#### Network meta-analysis

We will perform our network meta-analysis of diagnostic test accuracy using a Bayesian hierarchical framework with random effects using multiple models (15, 16). We will implement our analysis through a Markov Chain Monte Carlo simulation in WinBUGS, OpenBUGS, and R. Within our network diagram, the size of nodes will be proportional to the number of participants and the line connecting nodes will be proportional to the number of direct comparisons.

#### Pairwise meta-analysis

We will perform pairwise pooled analysis of diagnostic test characteristics, including sensitivity, specificity, likelihood ratios, diagnostic odds ratio, superiority index, and the area under the receiver operating curve. We will evaluate between-study heterogeneity using Cochrane Q and the inconsistency index (whereby *I*^2^ = 25%, 50%, and 75% will be indicative of low, moderate, and high statistical heterogeneity, respectively).

#### Indirect comparison and ranking of competing index tests

Our reference standard will be the resolution of hyperparathyroidism, as measured by circulating intact PTH level and/or corrected calcium level, at 3–6 months following surgery. Against this reference standard, we will be comparing the relative diagnostic outcomes for each index test. There remains controversy in the ideal pooled statistic for which to compare diagnostic test (17). Diagnostic odds ratio has been used to rank diagnostic tests, but its utility is hampered by its inability to weigh sensitivity and specificity independently and its lack of clinical applicability (18). An alternative statistic for ranking is the superiority index, which applies greater weight to index tests that perform well with both diagnostic test measures and lesser weight to those tests that perform well in one measure but poorly in another or perform poorly in both (19). Subgroup analysis may be challenging in the context of the chosen Bayesian network, but we will plan to perform sensitivity analyses, where applicable.

#### Publication bias analysis

If there are at least ten studies of the index test are included in the meta-analysis, then we will perform a Deeks' test of asymmetry and funnel plot analysis to evaluate for publication bias (20).

### Results reporting and presentation

We will report findings within the final publication in accordance with the PRISMA statement for diagnostic test accuracy meta-analysis (PRISMA-DTA) (21). A PRISMA flow diagram will be used for reporting the screening and article selection process, including the number of citations at each stage and the reasons for exclusion (i.e., full-text stage only). The discussion will include a summary of the major findings, the methodological limitations, and the application of these findings to clinical practice. The findings will be published in a peer-reviewed scientific journal and presented at national and/or international meetings. The published findings from the review will be disseminated to existing endocrine and otolaryngology–head and neck surgery networks.

## Discussion

This proposed meta-analysis has been registered in OSF and this protocol was developed to adhere to the PRISMA-P guidelines. Our proposed study will utilize pooled network analyses to determine the iPTH protocol and post-gland excision sampling timepoints most predictive of surgical cure in hyperparathyroidism. A network meta-analytic approach will permit direct and indirect comparisons and will facilitate ranking of iPTH criteria and, where applicable, post- excision sampling timepoints. These results will help to standardize the application of iPTH assays, optimize the use of these assays, and streamline healthcare expenditures in the management of hyperparathyroidism. Standardization of iPTH protocols may facilitate application in surgery for atypical parathyroid tumours and parathyroid carcinoma (22). In terms of possible final study limitations, there is the potential for inconsistent quality and selection bias in the reporting of included observational studies. We will employ a novel Bayesian statistical model to perform this network meta-analysis and provide a ranking of multiple diagnostic tests. Though hierarchical Bayesian models have been suggested for network meta-analysis, methodological challenges remain and so, development of our model will facilitate reproduction in future studies (23).

## Ethics statement

This study is exempt from institutional research board approval.

## Appendix A. Medline (Ovid) search strategy.

**Table:**

Search terms	No. citations
1. exp hyperparathyroidism.sh	13,671
2. exp hyperparathyroidism, primary.sh	3,842
3. exp hyperparathyroidism, secondary.sh	5,890
4. exp parathyroid hormone.sh	30,003
5. exp parathyroid neoplasms.sh	8,487
6. exp multiple endocrine neoplasia.sh	2,185
7. exp multiple endocrine neoplasia, type 1.sh	2,004
8. exp multiple endocrine neoplasia, type 2a.sh	1,421
9. exp hypoparathyroidism.sh	4,999
10. hyperparathyroid*.tw. or primary hyperparathyroid*.tw. or secondary hyperparathyroid*.tw. or tertiary hyperparathyroid*.tw. or parathyroid adenoma*.tw. or parathyroid hyperplas*.tw. or parathyroid multi-gland.tw. or (MEN 2 or multiple endocrine neoplasia type 2 or MEN type 2 or multiple endocrine neoplasia 2).tw. or (MEN 2a or multiple endocrine neoplasia type 2a or MEN type 2a or multiple endocrine neoplasia 2a).tw. or (MEN 1 or multiple endocrine neoplasia type 1 or MEN type 1 or multiple endocrine neoplasia 1).tw.	34,650
11. 1 or 2 or 3 or 4 or 5 or 6 or 7 or 8 or 9 or 10	63,808
12. limit 11 to (humans and yr = "1990 -Current”)	36,345
13. (Charleston criteria or Charleston protocol or (Vienna criteria or Vienna protocol) or (Rotterdam criteria or Rotterdam protocol) or parathyroid hormone monitor* or intraop* parathyroid hormone or intraop* PTH or pth monitor* or ioPTH or iPTH or Miami criteria or (Mayo criteria or Mayo protocol) or (Halle criteria or Halle protocol) or (Rome criteria or Rome protocol)).tw.	5,167
14. limited 13 to (humans and yr = ”1990 -Current”)	3,989
15. 12 and 14	2,351

## Appendix B. Data extraction form.

**Table:**

Study characteristics	
Study number	
First Author	
Year of publication	
Journal	
Location of study	
Study design	
Single or multi-centre	
Tertiary care centre (Y/N/NR)	
Patient characteristics	
Diagnosis (1 = PHPT, 2 = SHPT, 3 = THPT)	
No. patients enrolled	
No. patients analyzed	
No females (%)	
Relevant past medical history	
Length of follow up period	
Preoperative imaging	
Preoperative imaging performed (Y/N)	
Type of preoperative imaging (e.g., NM, US, NM + US, CT, None)	
Concordance between preoperative imaging modalities (Y/N) AND/OR preop localizing CT scan (Y/N)	
Preoperative imaging results	
Preoperative bloodwork	
Preoperative Ca (nadir or most recent value)	
Preoperative Cr (nadir or most recent value)	
Preoperative PTH (nadir or most recent value)	
Surgical details	
Primary vs. revision surgery	
Surgery technique (midline neck incision, video-assisted, minimally invasive)	
Central lab vs. point of care test for iPTH	
Central vs. peripheral blood sampling	
Baseline iPTH (pre-incision)	
Baseline iPTH (pre-ligation)	

**Table:**

Reference standard			
Index test (iPTH criteria)	Reference standard +	Reference standard −	Total
Name:			
Index test +	TP	FP	
Index test −	FN	TN	
Total			
Reference standard			
Index test (iPTH criteria)	Reference standard +	Reference standard −	Total
Name:			
Index test +	TP	FP	
Index test −	FN	TN	
Total			
Reference standard			
Index test (iPTH criteria)	Reference standard +	Reference standard −	Total
Name:			
Index test +	TP	FP	
Index test −	FN	TN	
Total			
Reference standard			
Index test (iPTH criteria)	Reference standard +	Reference standard −	Total
Name:			
Index test +	TP	FP	
Index test −	FN	TN	
Total			
Reference standard			
Index test (iPTH criteria)	Reference standard +	Reference standard −	Total
Name:			
Index test +	TP	FP	
Index test −	FN	TN	
Total			
Reference standard			
Index test (Post-excision timepoint)	Reference standard +	Reference standard −	Total
Name:			
Index test +	TP	FP	
Index test −	FN	TN	
Total			
Reference standard			
Index test (Post-excision timepoint)	Reference standard +	Reference standard −	Total
Name:			
Index test +	TP	FP	
Index test −	FN	TN	
Total			
Reference standard			
Index test (Post-excision timepoint)	Reference standard +	Reference standard −	Total
Name:			
Index test +	TP	FP	
Index test −	FN	TN	
Total			
Reference standard			
Index test (Post-excision timepoint)	Reference standard +	Reference standard −	Total
Name:			
Index test +	TP	FP	
Index test −	FN	TN	
Total			

**Table:**

Reference standard			
Index test (Post-excision timepoint)	Reference standard +	Reference standard −	Total
Name:			
Index test +	TP	FP	
Index test −	FN	TN	
Total			

TP, true positive; FP, false positive; FN, false negative; TN, true negative.
